# Biomedical Research Images Manipulated by Generative AI to Alter Scientific Outcomes: Diagnostic Study of Human and Automated Detection

**DOI:** 10.2196/100710

**Published:** 2026-10-02

**Authors:** Shuhang Luo, Runhua Tang, Ziyin Chen, Jianye Wang, Ming Liu, Jianfeng Wang, Li Ma

**Affiliations:** 1 Department of Urology, China-Japan Friendship Hospital, Beijing, Beijing, China; 2 Department of Urology, Beijing Hospital, Beijing, China, 8613911036970; 3 National Center for Gerontology, Beijing Hospital, Beijing, China; 4 National Clinical Research Center for Gerontology, Beijing Hospital, Beijing, China; 5 The Key Laboratory of Geriatrics of NHC, Beijing Hospital, Beijing, China; 6 Institute of Geriatric Medicine, Chinese Academy of Medical Sciences, Beijing Hospital, Beijing, China; 7 Department of Intensive Care Medicine, The Second Hospital & The Second Clinical Medical College of Lanzhou University, No. 82 CuiyingmenLanzhou, Gansu, 73003, China, 86 13893679654

**Keywords:** generative artificial intelligence, GenAI, GPT Images 2.0, biomedical image manipulation, conclusion-oriented falsification, research integrity, AI-generated image detection

## Abstract

In a diagnostic study of 104 western blot and subcutaneous xenograft tumor images, a high-fidelity generative model produced forgeries that could alter the conclusions of a study; 24 PhD-level expert reviewers could not reliably distinguish the forgeries from authentic figures (mean accuracy 50.5%, SD 6.9%), while the best-performing commercial AI detector achieved only moderate discrimination (area under the curve 0.790, 95% CI 0.695-0.885), revealing critical vulnerabilities in current research-integrity safeguards.

## Introduction

Generative AI (GenAI) has rapidly entered scientific workflows, lowering barriers to text and image creation [1]. While AI has shown strong performance in biomedical applications, its use in content generation raises major research integrity concerns, especially through the creation of plausible experimental results that are difficult to distinguish from authentic data [2-4]. As image-generation systems improve, it remains unclear whether they can produce manipulated biomedical research images able to alter the conclusions of a study in specific experimental settings and whether they are capable of misleading researchers and evading existing detection tools [5]. With the emergence of the high-fidelity model ChatGPT Images 2.0 (OpenAI), reassessing the risk of AI-enabled image falsification in biomedical manuscripts has become increasingly necessary. Here, we evaluated whether this system could create conclusion-altering images from authentic biomedical figures in 2 selected modalities—western blot and subcutaneous xenograft tumor images—and compared the performance of human readers with 3 commercial AI detection tools.

## Methods

### Overview

We assembled a benchmark dataset of 104 images, including 52 authentic images from 16 published biomedical studies and 52 matched AI-manipulated counterparts across western blot and subcutaneous xenograft tumor image subgroups (Figure 1). To simulate intentional falsification, the authentic images were input into ChatGPT Images 2.0 (accessed via the ChatGPT web interface on April 29, 2026) and iteratively refined using natural-language prompts (averaging 5‐10 attempts; eg, “preserve the background and reverse the protein trend”). Failed generations were discarded. The selected images were evaluated exactly as output, without manual postprocessing. Details of the selection workflow are provided in Multimedia Appendix 1. Each manipulated image preserved the visual characteristics of the source figure while altering the apparent direction of the experimental result. These image types were selected because they commonly serve as key visual evidence in biomedical manuscripts. Three commercial AI detection tools (Illuminarty, Is It AI, and AI or Not) selected for their high public accessibility despite being designed primarily for general images were evaluated in parallel with 24 PhD-level researchers with >5 years of experience (specializing in urology, oncology, and molecular biology). Only successfully generated and selected adversarial images were evaluated. As active manuscript peer reviewers highly familiar with these image types, they received zero prior calibration or forensic training in order to accurately simulate real-world peer-review vulnerabilities.

**Figure 1.**
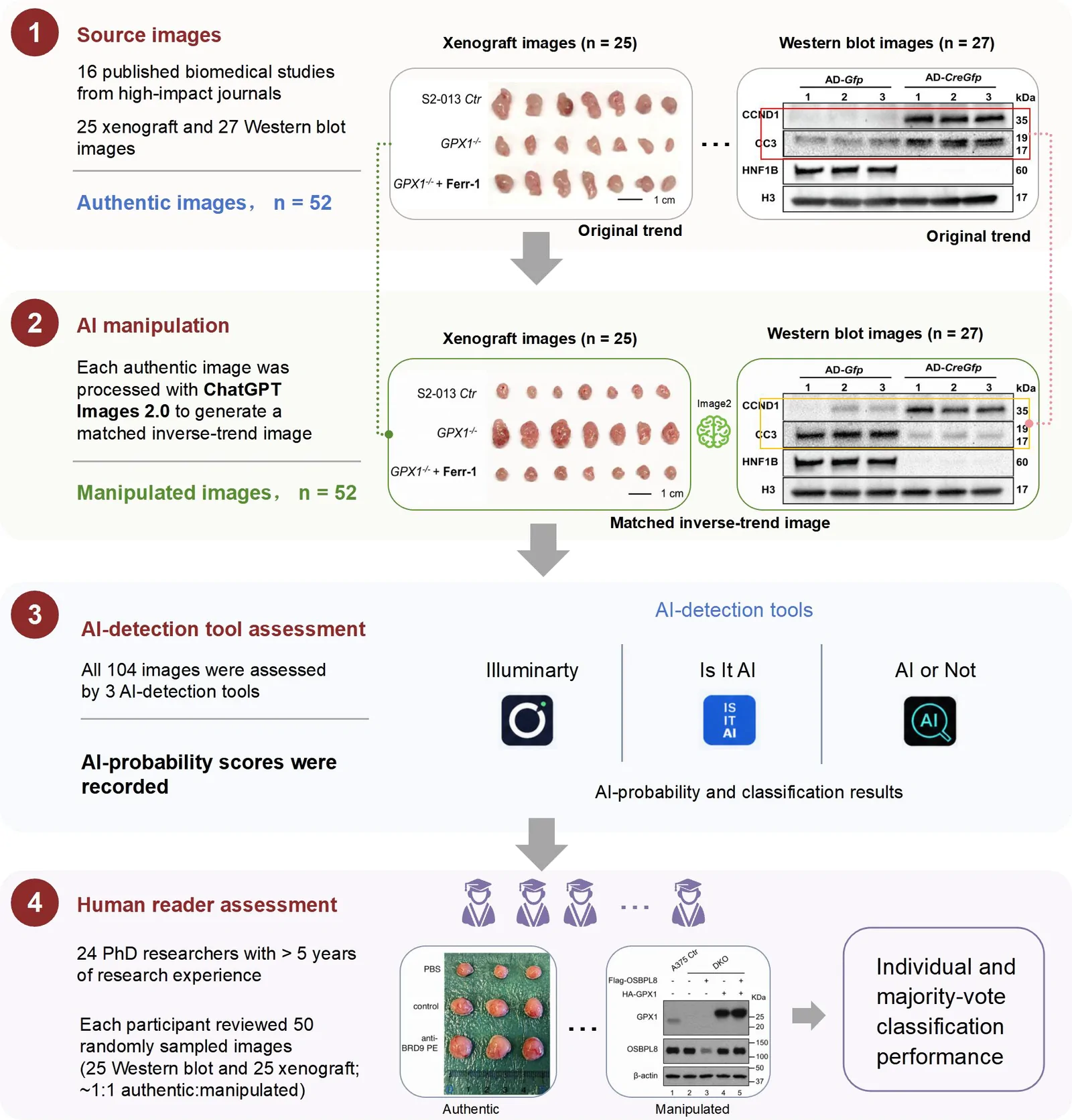
Workflow for generating and evaluating AI-manipulated biomedical research images.

### Ethical Considerations

The ethical approval for this study was granted by the Research Ethics Committee of Beijing Hospital (2021BJYYEC-030-01). Written informed consent was obtained from all participants.

## Results

Human evaluators showed limited ability to identify AI-manipulated images. Mean accuracy was 50.5% (SD 6.9%), with low sensitivity (38.5%, SD 18.9%) and modest specificity (61.5%, SD 17.7%; Figure 2A and B). Positive and negative predictive values were similarly limited, and the mean Youden index was 0, indicating near-chance performance. These findings suggest that among the evaluated western blot and xenograft images, the manipulated images retained sufficient visual credibility to evade routine expert judgment.

**Figure 2.**
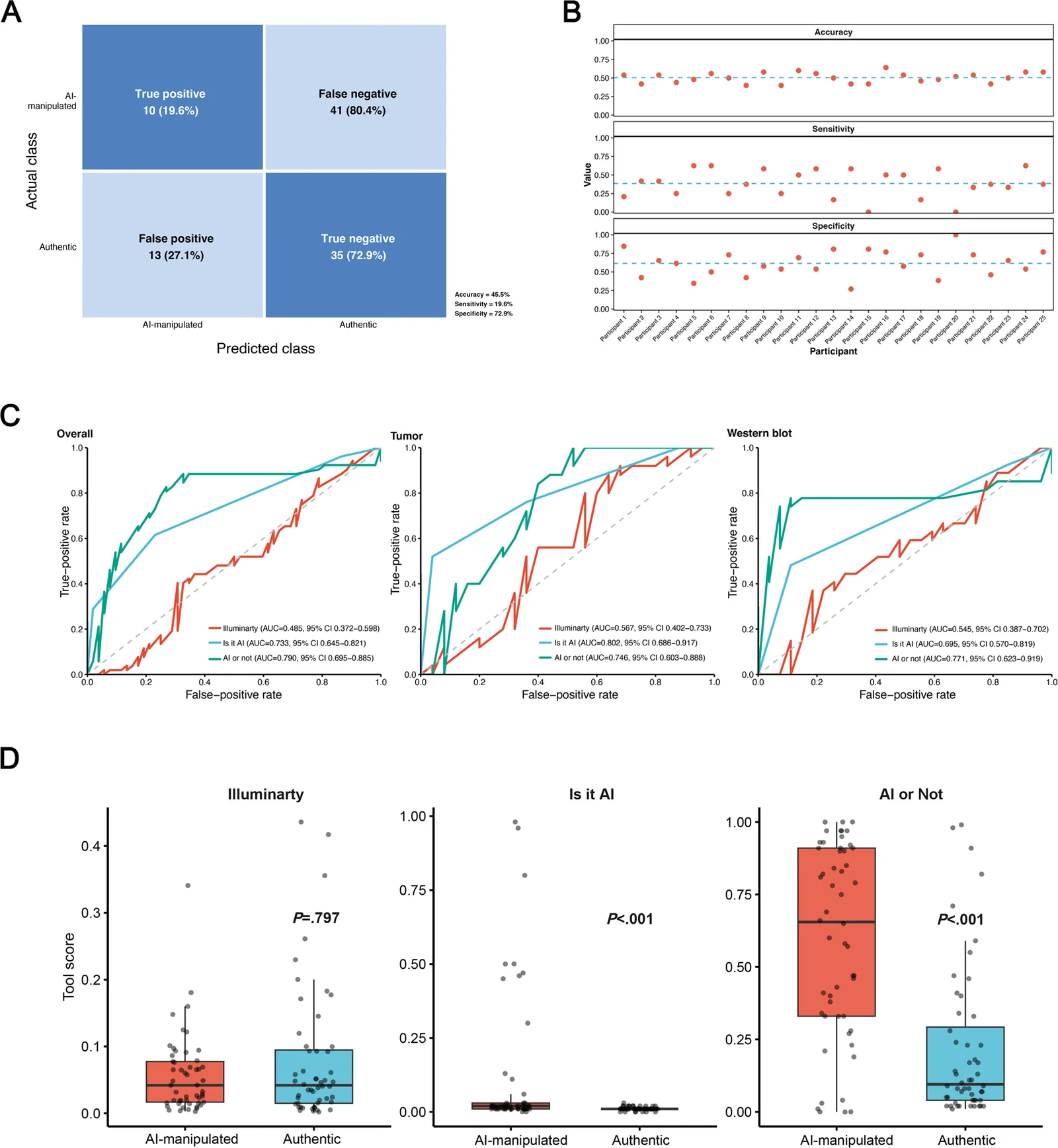
Human and automated detection of AI-manipulated scientific images.

Among the automated tools, AI or Not showed the best overall discrimination (area under the curve [AUC] 0.790, 95% CI 0.695‐0.885), followed by Is It AI (AUC 0.733, 95% CI 0.645‐0.821), whereas Illuminarty performed at near-chance level (AUC 0.485, 95% CI 0.372‐0.598; Figure 2C). AI or Not and Is It AI both significantly outperformed Illuminarty, whereas the difference between the first two was not significant. Performance varied by image type, but score distributions and confusion matrices showed the same overall pattern, with clearer separation for AI or Not and Is It AI (Figure 2D). Threshold-dependent analyses showed the expected trade-off between sensitivity and specificity, with the best overall balance at the Youden threshold. Paired and subgroup results were consistent with the main receiver operating curve findings and are summarized in Tables S4-S8 in Multimedia Appendix 1.

## Discussion

Previous studies have evaluated available AI detectors and concluded that they remain weak [6,7]. Despite continued advances in GenAI, our findings suggest that the practical utility of these tested tools remains limited for the types of images evaluated in this study. Although 2 tools showed moderate statistical discrimination, their performance was insufficient to provide reliable detection in the evaluated western blot and xenograft images. Furthermore, because proprietary commercial detectors are opaque and subject to unannounced updates, our findings represent a snapshot of their capabilities rather than a permanent evaluation. As editorial policies are still evolving [8], outputs from these tested detectors appear to have limited standalone value for research-integrity decision-making in this field.

Our findings add to concerns that AI detectors lag behind generative models [7,9]. False-positive classifications are particularly problematic [10], as they may trigger unnecessary investigation. Although some tools achieved AUCs above 0.7, their performance remained insufficient for dependable deployment in the evaluated types of image, leaving no clearly actionable threshold for routine use. More importantly, researchers with substantial scientific training were unable to reliably distinguish authentic from AI-manipulated images in the evaluated western blot and xenograft images. Thus, neither domain expertise nor the tested commercially available detectors provided a robust safeguard against manipulations optimized for visual plausibility.

Unlike traditional image falsification, which often requires technical editing skills and may leave detectable artifacts, GenAI can allow users to alter visual evidence through natural-language prompts while preserving overall visual coherence, as demonstrated in the evaluated western blot and xenograft examples. This may lower the barrier to fabrication and make problematic images harder to detect during routine review.

This proof-of-concept study has several limitations. First, external validity is limited because the dataset was small (n=104) and restricted to 2 image modalities—western blot and subcutaneous xenograft tumor images—derived from 52 source images across 16 published studies. These findings therefore should not be generalized broadly to biomedical research images but interpreted within the specific image types evaluated here. Second, the benchmark included only successfully generated outputs and excluded failed attempts; thus, the reported metrics are conditional on successful manipulation and do not represent the overall probability of generating an undetectable forgery from a given source image. Third, the human-evaluator experiment had a clustered structure, as each participant reviewed multiple images and each image was assessed by multiple participants. Pooled reader responses were therefore not statistically independent and should be interpreted descriptively; a more rigorous inferential analysis would require a mixed effects or multi-evaluator, multicase framework. Future studies should expand to additional biomedical image types, figure formats, evaluators, and generation scenarios.

The key implication is that current GenAI can enable conclusion-altering falsification in at least some biomedical image types, including the western blot and xenograft examples evaluated here. This risk is concerning because conclusion-altering falsification in biomedical manuscripts could misdirect subsequent research and waste substantial resources if not identified. Historical cases of experts publishing fabricated data in top-tier journals underscore the devastating downstream effects of such misconduct.

To counter these emerging GenAI-related risks, particularly in image-based biomedical submissions, we propose a practical, 3-step editorial workflow. First, at submission, journals must mandate original, uncropped raw images (eg, full-length membranes, time-stamped photos). Second, during editorial triage, staff should routinely screen image metadata (eg, EXIF data), immediately flagging stripped provenance. Third, peer reviewers should be instructed to shift their focus from traditional digital artifacts to biological inconsistencies (eg, anomalous molecular weight alignments or unphysiological growth rates) that GenAI frequently produces.

## Supplementary material

10.2196/100710 Multimedia Appendix 1 Supplementary information on methods, with tables and figures showing extended data.

## Abbreviations

AUC: area under the curve
GenAI: generative AI
