# Supplementary material for: Biomedical Research Images Manipulated by Generative AI to Alter Scientific Outcomes: Diagnostic Study of Human and Automated Detection
Source: J Med Internet Res. 2026 Oct 2;28:e100710. doi: 10.2196/100710 (PMC13633064; doi:10.2196/100710)
Supplement: Supplementary file 1 [file jmir-v28-e100710-s001.docx]

**Extended Data Table 1** Source information, image identifiers, ground-truth labels and AI-generation probabilities assigned by three detection tools

| Source article number | Journal | PMID | Image Type | Ground-truth label | Image ID | Illuminarty AI probability (%) | Is It AI probability (%) | AI-or-Not probability (%) |
| --- | --- | --- | --- | --- | --- | --- | --- | --- |
| 1 | Nature | 26375259 | Western blot | Authentic | WT1 | 2.4% | 1.0% | 4.0% |
| 1 | Nature | 26375259 | Western blot | Authentic | WT2 | 2.7% | 1.0% | 23.0% |
| 1 | Nature | 26375259 | Western blot | Authentic | WT3 | 0.4% | 1.0% | 4.0% |
| 1 | Nature | 26375259 | Western blot | AI-manipulated | WF1 | 2.6% | 1.0% | 0.0% |
| 1 | Nature | 26375259 | Western blot | AI-manipulated | WF2 | 6.0% | 2.0% | 100.0% |
| 1 | Nature | 26375259 | Western blot | AI-manipulated | WF3 | 7.7% | 1.0% | 0.0% |
| 2 | Nature | 22722839 | Western blot | Authentic | WT4 | 0.8% | 1.0% | 13.0% |
| 2 | Nature | 22722839 | Western blot | Authentic | WT5 | 0.9% | 1.0% | 18.0% |
| 2 | Nature | 22722839 | Western blot | AI-manipulated | WF4 | 0.9% | 2.0% | 100.0% |
| 2 | Nature | 22722839 | Western blot | AI-manipulated | WF5 | 4.9% | 1.0% | 100.0% |
| 3 | Nature | 24695230 | Western blot | Authentic | WT6 | 4.8% | 1.0% | 4.0% |
| 3 | Nature | 24695230 | Western blot | AI-manipulated | WF6 | 3.2% | 3.0% | 0.0% |
| 4 | Cell | 41406964 | Western blot | Authentic | WT7 | 2.3% | 0.0% | 8.0% |
| 4 | Cell | 41406964 | Western blot | Authentic | WT8 | 5.2% | 0.0% | 2.0% |
| 4 | Cell | 41406964 | Western blot | Authentic | WT9 | 4.7% | 1.0% | 2.0% |
| 4 | Cell | 41406964 | Western blot | Authentic | WT10 | 23.0% | 0.0% | 7.0% |
| 4 | Cell | 41406964 | Western blot | Authentic | WT11 | 18.3% | 0.0% | 11.0% |
| 4 | Cell | 41406964 | Western blot | AI-manipulated | WF7 | 1.7% | 80.0% | 85.0% |
| 4 | Cell | 41406964 | Western blot | AI-manipulated | WF8 | 1.4% | 2.0% | 27.0% |
| 4 | Cell | 41406964 | Western blot | AI-manipulated | WF9 | 12.5% | 2.0% | 57.0% |
| 4 | Cell | 41406964 | Western blot | AI-manipulated | WF10 | 14.8% | 2.0% | 1.0% |
| 4 | Cell | 41406964 | Western blot | AI-manipulated | WF11 | 8.6% | 0.0% | 65.0% |
| 5 | Cell | 41720096 | Western blot | Authentic | WT12 | 4.1% | 1.0% | 5.0% |
| 5 | Cell | 41720096 | Western blot | Authentic | WT13 | 1.4% | 1.0% | 4.0% |
| 5 | Cell | 41720096 | Western blot | Authentic | WT14 | 0.3% | 1.0% | 34.0% |
| 5 | Cell | 41720096 | Western blot | Authentic | WT15 | 4.9% | 1.0% | 7.0% |
| 5 | Cell | 41720096 | Western blot | Authentic | WT16 | 0.5% | 2.0% | 82.0% |
| 5 | Cell | 41720096 | Western blot | AI-manipulated | WF12 | 6.5% | 1.0% | 75.0% |
| 5 | Cell | 41720096 | Western blot | AI-manipulated | WF13 | 1.6% | 2.0% | 84.0% |
| 5 | Cell | 41720096 | Western blot | AI-manipulated | WF14 | 0.7% | 1.0% | 91.0% |
| 5 | Cell | 41720096 | Western blot | AI-manipulated | WF15 | 4.2% | 2.0% | 91.0% |
| 5 | Cell | 41720096 | Western blot | AI-manipulated | WF16 | 1.4% | 2.0% | 82.0% |
| 5 | Cell | 41720096 | Xenograft | Authentic | TT4 | 26.1% | 2.0% | 2.0% |
| 5 | Cell | 41720096 | Xenograft | Authentic | TT5 | 5.1% | 1.0% | 23.0% |
| 5 | Cell | 41720096 | Xenograft | Authentic | TT6 | 12.0% | 1.0% | 2.0% |
| 5 | Cell | 41720096 | Xenograft | Authentic | TT7 | 17.7% | 0.0% | 2.0% |
| 5 | Cell | 41720096 | Xenograft | AI-manipulated | TF4 | 10.1% | 50.0% | 46.0% |
| 5 | Cell | 41720096 | Xenograft | AI-manipulated | TF5 | 6.9% | 13.0% | 23.0% |
| 5 | Cell | 41720096 | Xenograft | AI-manipulated | TF6 | 6.5% | 46.0% | 33.0% |
| 5 | Cell | 41720096 | Xenograft | AI-manipulated | TF7 | 6.5% | 96.0% | 97.0% |
| 6 | Science | 41926552 | Western blot | Authentic | WT17 | 10.0% | 2.0% | 8.0% |
| 6 | Science | 41926552 | Western blot | Authentic | WT18 | 9.3% | 1.0% | 17.0% |
| 6 | Science | 41926552 | Western blot | Authentic | WT19 | 6.3% | 2.0% | 9.0% |
| 6 | Science | 41926552 | Western blot | AI-manipulated | WF17 | 16.0% | 2.0% | 4.0% |
| 6 | Science | 41926552 | Western blot | AI-manipulated | WF18 | 9.1% | 1.0% | 90.0% |
| 6 | Science | 41926552 | Western blot | AI-manipulated | WF19 | 4.2% | 2.0% | 93.0% |
| 7 | Science | 41747053 | Western blot | Authentic | WT20 | 5.8% | 1.0% | 8.0% |
| 7 | Science | 41747053 | Western blot | Authentic | WT21 | 4.6% | 1.0% | 91.0% |
| 7 | Science | 41747053 | Western blot | Authentic | WT22 | 0.7% | 1.0% | 10.0% |
| 7 | Science | 41747053 | Western blot | Authentic | WT23 | 6.1% | 1.0% | 2.0% |
| 7 | Science | 41747053 | Western blot | Authentic | WT24 | 3.8% | 1.0% | 3.0% |
| 7 | Science | 41747053 | Western blot | AI-manipulated | WF20 | 5.8% | 1.0% | 91.0% |
| 7 | Science | 41747053 | Western blot | AI-manipulated | WF21 | 1.2% | 1.0% | 3.0% |
| 7 | Science | 41747053 | Western blot | AI-manipulated | WF22 | 0.5% | 1.0% | 97.0% |
| 7 | Science | 41747053 | Western blot | AI-manipulated | WF23 | 9.3% | 1.0% | 93.0% |
| 7 | Science | 41747053 | Western blot | AI-manipulated | WF24 | 12.2% | 2.0% | 95.0% |
| 8 | Science | 41990178 | Western blot | Authentic | WT25 | 2.0% | 1.0% | 9.0% |
| 8 | Science | 41990178 | Western blot | Authentic | WT26 | 41.7% | 1.0% | 1.0% |
| 8 | Science | 41990178 | Western blot | Authentic | WT27 | 3.5% | 1.0% | 2.0% |
| 8 | Science | 41990178 | Western blot | AI-manipulated | WF25 | 4.8% | 1.0% | 78.0% |
| 8 | Science | 41990178 | Western blot | AI-manipulated | WF26 | 7.9% | 1.0% | 58.0% |
| 8 | Science | 41990178 | Western blot | AI-manipulated | WF27 | 0.4% | 0.0% | 97.0% |
| 9 | Cell | 41406964 | Xenograft | Authentic | TT1 | 1.7% | 1.0% | 46.0% |
| 9 | Cell | 41406964 | Xenograft | Authentic | TT2 | 9.3% | 1.0% | 14.0% |
| 9 | Cell | 41406964 | Xenograft | Authentic | TT3 | 14.5% | 1.0% | 7.0% |
| 9 | Cell | 41406964 | Xenograft | AI-manipulated | TF1 | 1.9% | 1.0% | 38.0% |
| 9 | Cell | 41406964 | Xenograft | AI-manipulated | TF2 | 1.9% | 1.0% | 41.0% |
| 9 | Cell | 41406964 | Xenograft | AI-manipulated | TF3 | 2.5% | 2.0% | 81.0% |
| 10 | Science Advances | 42030388 | Xenograft | Authentic | TT8 | 4.3% | 1.0% | 6.0% |
| 10 | Science Advances | 42030388 | Xenograft | Authentic | TT9 | 2.2% | 1.0% | 2.0% |
| 10 | Science Advances | 42030388 | Xenograft | Authentic | TT10 | 0.2% | 0.0% | 17.0% |
| 10 | Science Advances | 42030388 | Xenograft | AI-manipulated | TF8 | 2.6% | 98.0% | 97.0% |
| 10 | Science Advances | 42030388 | Xenograft | AI-manipulated | TF9 | 0.8% | 45.0% | 43.0% |
| 10 | Science Advances | 42030388 | Xenograft | AI-manipulated | TF10 | 34.1% | 2.0% | 34.0% |
| 11 | Nature | 40500442 | Xenograft | Authentic | TT11 | 4.0% | 1.0% | 5.0% |
| 11 | Nature | 40500442 | Xenograft | Authentic | TT12 | 0.9% | 3.0% | 24.0% |
| 11 | Nature | 40500442 | Xenograft | AI-manipulated | TF11 | 6.9% | 1.0% | 21.0% |
| 11 | Nature | 40500442 | Xenograft | AI-manipulated | TF12 | 1.0% | 30.0% | 19.0% |
| 12 | Cell death and differentiation | 39972058 | Xenograft | Authentic | TT13 | 35.6% | 1.0% | 2.0% |
| 12 | Cell death and differentiation | 39972058 | Xenograft | AI-manipulated | TF13 | 1.9% | 1.0% | 33.0% |
| 13 | Nature | [39112700](https://pubmed.ncbi.nlm.nih.gov/39112700/) | Xenograft | Authentic | TT14 | 0.9% | 0.0% | 13.0% |
| 13 | Nature | [39112700](https://pubmed.ncbi.nlm.nih.gov/39112700/) | Xenograft | Authentic | TT15 | 1.5% | 1.0% | 40.0% |
| 13 | Nature | [39112700](https://pubmed.ncbi.nlm.nih.gov/39112700/) | Xenograft | AI-manipulated | TF14 | 1.5% | 11.0% | 33.0% |
| 13 | Nature | [39112700](https://pubmed.ncbi.nlm.nih.gov/39112700/) | Xenograft | AI-manipulated | TF15 | 0.3% | 47.0% | 40.0% |
| 14 | Nature Biotechnology | 39905264 | Xenograft | Authentic | TT16 | 3.3% | 2.0% | 71.0% |
| 14 | Nature Biotechnology | 39905264 | Xenograft | Authentic | TT17 | 43.6% | 2.0% | 55.0% |
| 14 | Nature Biotechnology | 39905264 | Xenograft | Authentic | TT18 | 0.5% | 2.0% | 99.0% |
| 14 | Nature Biotechnology | 39905264 | Xenograft | Authentic | TT19 | 9.2% | 2.0% | 98.0% |
| 14 | Nature Biotechnology | 39905264 | Xenograft | AI-manipulated | TF16 | 9.4% | 2.0% | 90.0% |
| 14 | Nature Biotechnology | 39905264 | Xenograft | AI-manipulated | TF17 | 9.7% | 2.0% | 60.0% |
| 14 | Nature Biotechnology | 39905264 | Xenograft | AI-manipulated | TF18 | 1.9% | 6.0% | 79.0% |
| 14 | Nature Biotechnology | 39905264 | Xenograft | AI-manipulated | TF19 | 2.9% | 3.0% | 92.0% |
| 15 | Cell death discovery | 41057306 | Xenograft | Authentic | TT20 | 17.1% | 2.0% | 47.0% |
| 15 | Cell death discovery | 41057306 | Xenograft | AI-manipulated | TF20 | 6.6% | 2.0% | 47.0% |
| 16 | Nature | 31578522 | Xenograft | Authentic | TT21 | 20.0% | 1.0% | 28.0% |
| 16 | Nature | 31578522 | Xenograft | Authentic | TT22 | 1.5% | 1.0% | 11.0% |
| 16 | Nature | 31578522 | Xenograft | AI-manipulated | TF21 | 7.6% | 50.0% | 28.0% |
| 16 | Nature | 31578522 | Xenograft | AI-manipulated | TF22 | 18.1% | 2.0% | 47.0% |
| 17 | Nature | 31597964 | Xenograft | Authentic | TT23 | 0.9% | 2.0% | 59.0% |
| 17 | Nature | 31597964 | Xenograft | Authentic | TT24 | 10.0% | 1.0% | 41.0% |
| 17 | Nature | 31597964 | Xenograft | Authentic | TT25 | 3.5% | 2.0% | 33.0% |
| 17 | Nature | 31597964 | Xenograft | AI-manipulated | TF23 | 0.6% | 1.0% | 69.0% |
| 17 | Nature | 31597964 | Xenograft | AI-manipulated | TF24 | 2.3% | 1.0% | 66.0% |
| 17 | Nature | 31597964 | Xenograft | AI-manipulated | TF25 | 3.3% | 3.0% | 83.0% |

**Extended Data Table 2** Diagnostic performance of three AI-detection tools for identifying AI-manipulated images in the overall dataset and in image-specific subgroups

| Dataset | Tool | AUC (95% CI) | Threshold | Sensitivity | Specificity | PPV | NPV | Accuracy | F1 score | Youden index | LR+ | LR− |
| --- | --- | --- | --- | --- | --- | --- | --- | --- | --- | --- | --- | --- |
| Overall | Illuminarty | 0.485 (0.372–0.598) | 0.064 | 0.404 | 0.692 | 0.568 | 0.537 | 0.548 | 0.472 | 0.096 | 1.312 | 0.861 |
| Overall | Is It AI | 0.733 (0.645–0.821) | 0.015 | 0.615 | 0.769 | 0.727 | 0.667 | 0.692 | 0.667 | 0.385 | 2.667 | 0.500 |
| Overall | AI or Not | 0.790 (0.695–0.885) | 0.185 | 0.885 | 0.673 | 0.730 | 0.854 | 0.779 | 0.800 | 0.558 | 2.706 | 0.171 |
| Western blot | Illuminarty | 0.545 (0.387–0.702) | 0.064 | 0.370 | 0.815 | 0.667 | 0.564 | 0.593 | 0.476 | 0.185 | 2.000 | 0.773 |
| Western blot | Is It AI | 0.695 (0.570–0.819) | 0.015 | 0.481 | 0.889 | 0.812 | 0.632 | 0.685 | 0.605 | 0.370 | 4.333 | 0.583 |
| Western blot | AI or Not | 0.771 (0.623–0.919) | 0.250 | 0.778 | 0.889 | 0.875 | 0.800 | 0.833 | 0.824 | 0.667 | 7.000 | 0.250 |
| Tumor | Illuminarty | 0.567 (0.402–0.733) | 0.110 | 0.920 | 0.320 | 0.575 | 0.800 | 0.620 | 0.708 | 0.240 | 1.353 | 0.250 |
| Tumor | Is It AI | 0.802 (0.686–0.917) | 0.025 | 0.520 | 0.960 | 0.929 | 0.667 | 0.740 | 0.667 | 0.480 | 13.000 | 0.500 |
| Tumor | AI or Not | 0.746 (0.603–0.888) | 0.180 | 1.000 | 0.480 | 0.658 | 1.000 | 0.740 | 0.794 | 0.480 | 1.923 | 0.000 |

Abbreviations: AUC, area under the receiver operating characteristic curve; CI, confidence interval; PPV, positive predictive value; NPV, negative predictive value; LR+, positive likelihood ratio; LR−, negative likelihood ratio.

**Extended Data Table 3** Pairwise comparisons of AUCs using DeLong’s test

| Dataset | Comparison | AUC 1 | AUC 2 | P value |
| --- | --- | --- | --- | --- |
| Overall | Illuminarty vs Is It AI | 0.485 | 0.733 | 0.0005 |
| Overall | Illuminarty vs AI or Not | 0.485 | 0.790 | 0.0001 |
| Overall | Is It AI vs AI or Not | 0.733 | 0.790 | 0.3398 |
| Western blot | Illuminarty vs Is It AI | 0.545 | 0.695 | 0.1530 |
| Western blot | Illuminarty vs AI or Not | 0.545 | 0.771 | 0.0514 |
| Western blot | Is It AI vs AI or Not | 0.695 | 0.771 | 0.4466 |
| Xenograft | Illuminarty vs Is It AI | 0.567 | 0.802 | 0.0254* |
| Xenograft | Illuminarty vs AI or Not | 0.567 | 0.746 | 0.0932* |
| Xenograft | Is It AI vs AI or Not | 0.802 | 0.746 | 0.4786 |

* Comparisons involving Illuminarty in the tumor subgroup should be interpreted with caution because the ROC curves were generated with different score directions.

**Extended Data Table 4** Overall diagnostic performance of the three AI-image detection tools

| Tool | AUC  (95% CI) | Bootstrap AUC (95% CI) | Threshold strategy | Threshold | Sensitivity | Specificity | PPV | NPV | Accuracy |
| --- | --- | --- | --- | --- | --- | --- | --- | --- | --- |
| Illuminarty | 0.485 (0.372–0.598) | 0.486 (0.403–0.577) | Default (0.5) | 0.500 | 0.000 | 1.000 | - | 0.500 | 0.500 |
| Illuminarty | 0.485 (0.372–0.598) | 0.486 (0.403–0.577) | Youden | 0.064 | 0.404 | 0.692 | 0.568 | 0.537 | 0.548 |
| Illuminarty | 0.485 (0.372–0.598) | 0.486 (0.403–0.577) | Specificity ≈95% | 0.387 | 0.000 | 0.962 | 0.000 | 0.490 | 0.481 |
| Is It AI | 0.733 (0.645–0.821) | 0.733 (0.663–0.800) | Default (0.5) | 0.500 | 0.096 | 1.000 | 1.000 | 0.525 | 0.548 |
| Is It AI | 0.733 (0.645–0.821) | 0.733 (0.663–0.800) | Youden | 0.015 | 0.615 | 0.769 | 0.727 | 0.667 | 0.692 |
| Is It AI | 0.733 (0.645–0.821) | 0.733 (0.663–0.800) | Specificity ≈95% | 0.025 | 0.288 | 0.981 | 0.938 | 0.580 | 0.635 |
| AI or Not | 0.790 (0.695–0.885) | 0.790 (0.701–0.878) | Default (0.5) | 0.500 | 0.577 | 0.865 | 0.811 | 0.672 | 0.721 |
| AI or Not | 0.790 (0.695–0.885) | 0.790 (0.701–0.878) | Youden | 0.185 | 0.885 | 0.673 | 0.730 | 0.854 | 0.779 |
| AI or Not | 0.790 (0.695–0.885) | 0.790 (0.701–0.878) | Specificity ≈95% | 0.915 | 0.212 | 0.962 | 0.846 | 0.549 | 0.587 |

Note: PPV and NPV shown here are based on the study sample prevalence of 50%. Abbreviation: PPV, positive predictive value; NPV, negative predictive value.

**Extended Data Table 5** ROC discrimination performance and DeLong pairwise comparisons

| Dataset | Tool | N | AUC | 95% CI | Bootstrap mean AUC | Bootstrap 95% CI |
| --- | --- | --- | --- | --- | --- | --- |
| Overall | Illuminarty | 104 | 0.485 | 0.372–0.598 | 0.486 | 0.403–0.577 |
| Overall | Is It AI | 104 | 0.733 | 0.645–0.821 | 0.733 | 0.663–0.800 |
| Overall | AI or Not | 104 | 0.790 | 0.695–0.885 | 0.790 | 0.701–0.878 |
| Western blot | Illuminarty | 54 | 0.545 | 0.387–0.702 | 0.543 | 0.441–0.650 |
| Western blot | Is It AI | 54 | 0.695 | 0.570–0.819 | 0.693 | 0.582–0.803 |
| Western blot | AI or Not | 54 | 0.771 | 0.623–0.919 | 0.774 | 0.619–0.913 |
| Xenograft | Illuminarty | 50 | 0.433 | 0.267–0.598 | 0.433 | 0.286–0.580 |
| Xenograft | Is It AI | 50 | 0.802 | 0.686–0.917 | 0.799 | 0.685–0.899 |
| Xenograft | AI or Not | 50 | 0.746 | 0.603–0.888 | 0.745 | 0.641–0.849 |

**Extended Data Table 6** Pairwise DeLong tests

| Dataset | Comparison | P-value |
| --- | --- | --- |
| Overall | Illuminarty vs. Is It AI | 0.00052 |
| Overall | Illuminarty vs. AI or Not | 0.00012 |
| Overall | Is It AI vs. AI or Not | 0.33977 |
| Western blot | Illuminarty vs. Is It AI | 0.15299 |
| Western blot | Illuminarty vs. AI or Not | 0.05137 |
| Western blot | Is It AI vs. AI or Not | 0.44661 |
| Xenograft | Illuminarty vs. Is It AI | 0.00026 |
| Xenograft | Illuminarty vs. AI or Not | 0.00723 |
| Xenograft | Is It AI vs. AI or Not | 0.47856 |

**Extended Data Table 7** Threshold-dependent diagnostic performance overall and by image modality

| Dataset | Tool | Threshold strategy | Threshold | Sensitivity | Specificity | Accuracy | PPV | NPV | F1 | Youden | LR+ | LR- |
| --- | --- | --- | --- | --- | --- | --- | --- | --- | --- | --- | --- | --- |
| Overall | Illuminarty | Default 0.5 | 0.500 | 0.000 | 1.000 | 0.500 | NA | 0.500 | 0.000 | 0.000 | NA | 1.000 |
| Overall | Illuminarty | Youden | 0.064 | 0.404 | 0.692 | 0.548 | 0.568 | 0.537 | 0.472 | 0.096 | 1.313 | 0.861 |
| Overall | Illuminarty | Specificity ≈95% | 0.387 | 0.000 | 0.962 | 0.481 | 0.000 | 0.490 | 0.000 | -0.038 | 0.000 | 1.040 |
| Overall | Is It AI | Default 0.5 | 0.500 | 0.096 | 1.000 | 0.548 | 1.000 | 0.525 | 0.175 | 0.096 | NA | 0.904 |
| Overall | Is It AI | Youden | 0.015 | 0.615 | 0.769 | 0.692 | 0.727 | 0.667 | 0.667 | 0.385 | 2.667 | 0.500 |
| Overall | Is It AI | Specificity ≈95% | 0.025 | 0.288 | 0.981 | 0.635 | 0.938 | 0.580 | 0.441 | 0.269 | 15.000 | 0.725 |
| Overall | AI or Not | Default 0.5 | 0.500 | 0.577 | 0.865 | 0.721 | 0.811 | 0.672 | 0.674 | 0.442 | 4.286 | 0.489 |
| Overall | AI or Not | Youden | 0.185 | 0.885 | 0.673 | 0.779 | 0.730 | 0.854 | 0.800 | 0.558 | 2.706 | 0.171 |
| Overall | AI or Not | Specificity ≈95% | 0.915 | 0.212 | 0.962 | 0.587 | 0.846 | 0.549 | 0.338 | 0.173 | 5.500 | 0.820 |
| Western blot | Illuminarty | Default 0.5 | 0.500 | 0.000 | 1.000 | 0.500 | NA | 0.500 | 0.000 | 0.000 | NA | 1.000 |
| Western blot | Illuminarty | Youden | 0.064 | 0.370 | 0.815 | 0.593 | 0.667 | 0.564 | 0.476 | 0.185 | 2.000 | 0.773 |
| Western blot | Illuminarty | Specificity ≈95% | 0.324 | 0.000 | 0.963 | 0.481 | 0.000 | 0.491 | 0.000 | -0.037 | 0.000 | 1.038 |
| Western blot | Is It AI | Default 0.5 | 0.500 | 0.037 | 1.000 | 0.519 | 1.000 | 0.509 | 0.071 | 0.037 | NA | 0.963 |
| Western blot | Is It AI | Youden | 0.015 | 0.481 | 0.889 | 0.685 | 0.813 | 0.632 | 0.605 | 0.370 | 4.333 | 0.583 |
| Western blot | Is It AI | Specificity ≈95% | 0.025 | 0.074 | 1.000 | 0.537 | 1.000 | 0.519 | 0.138 | 0.074 | NA | 0.926 |
| Western blot | AI or Not | Default 0.5 | 0.500 | 0.741 | 0.926 | 0.833 | 0.909 | 0.781 | 0.816 | 0.667 | 10.000 | 0.280 |
| Western blot | AI or Not | Youden | 0.250 | 0.778 | 0.889 | 0.833 | 0.875 | 0.800 | 0.824 | 0.667 | 7.000 | 0.250 |
| Western blot | AI or Not | Specificity ≈95% | 0.830 | 0.519 | 0.963 | 0.741 | 0.933 | 0.667 | 0.667 | 0.481 | 14.000 | 0.500 |
| Xenograft | Illuminarty | Default (0.5) | 0.500 | 0.000 | 1.000 | 0.500 | NA | 0.500 | 0.000 | 0.000 | NA | 1.000 |
| Xenograft | Illuminarty | Youden | 0.018 | 0.800 | 0.320 | 0.560 | 0.541 | 0.615 | 0.645 | 0.120 | 1.176 | 0.625 |
| Xenograft | Illuminarty | Specificity ≈95% | 0.396 | 0.000 | 0.960 | 0.480 | 0.000 | 0.490 | 0.000 | -0.040 | 0.000 | 1.042 |
| Xenograft | Is It AI | Default (0.5) | 0.500 | 0.160 | 1.000 | 0.580 | 1.000 | 0.543 | 0.276 | 0.160 | NA | 0.840 |
| Xenograft | Is It AI | Youden | 0.025 | 0.520 | 0.960 | 0.740 | 0.929 | 0.667 | 0.667 | 0.480 | 13.000 | 0.500 |
| Xenograft | Is It AI | Specificity ≈95% | 0.025 | 0.520 | 0.960 | 0.740 | 0.929 | 0.667 | 0.667 | 0.480 | 13.000 | 0.500 |
| Xenograft | AI or Not | Default (0.5) | 0.500 | 0.400 | 0.800 | 0.600 | 0.667 | 0.571 | 0.500 | 0.200 | 2.000 | 0.750 |
| Xenograft | AI or Not | Youden | 0.180 | 1.000 | 0.480 | 0.740 | 0.658 | 1.000 | 0.794 | 0.480 | 1.923 | 0.000 |
| Xenograft | AI or Not | Specificity ≈95% | 0.985 | 0.000 | 0.960 | 0.480 | 0.000 | 0.490 | 0.000 | -0.040 | 0.000 | 1.042 |

**Extended Data Table 8** Paired Wilcoxon signed-rank test results

| Dataset | Tool | V statistic | P value | Median paired difference |
| --- | --- | --- | --- | --- |
| Overall | Illuminarty | 516.0 | 0.91661 | -0.002 |
| Overall | Is It AI | 391.0 | 0.0000088 | 0.005 |
| Overall | AI or Not | 1032.0 | 0.00000038 | 0.335 |
| Western blot | Illuminarty | 183.5 | 0.42446 | 0.001 |
| Western blot | Is It AI | 62.0 | 0.00455 | 0.000 |
| Western blot | AI or Not | 313.0 | 0.00025 | 0.700 |
| Xenograft | Illuminarty | 96.5 | 0.96322 | -0.014 |
| Xenograft | Is It AI | 149.0 | 0.00032 | 0.010 |
| Xenograft | AI or Not | 213.0 | 0.00037 | 0.190 |

**Extended Data Figure 1** Comparison of AI-detection scores for AI-manipulated and authentic images across tumor and western blot subgroups.

Box plots show the distribution of scores from Illuminartt, Is It AI and AI or No for AI-manipulated and authentic images in the tumor and western blot subgroups. Each point represents one image. Center lines indicate medians, box limits indicate the interquartile range (IQR), and whiskers extend to 1.5 × IQR. P values were calculated using two-sided Wilcoxon rank-sum tests to compare AI-manipulated and authentic images within each subgroup.

**Extended Data Figure 2** Confusion matrices for three AI-detection tools applied to AI-manipulated and authentic images.

Matrices show true versus predicted classes for AI-manipulated and authentic images across the three AI-detection tools (AI or Not, Illuminarty, Is It AI). Each panel represents a tool, and each block indicates the number of true positives (TP), false positives (FP), true negatives (TN), and false negatives (FN). Rows correspond to the actual class and columns correspond to the predicted class. Separate sections represent overall, tumor, and Western blot image subgroups. These matrices summarize each tool’s classification performance and allow comparison of accuracy across tools and image subgroups.

# Methods

## Study design

We assembled a paired image dataset comprising 104 biomedical images, including 52 authentic images and 52 AI-manipulated counterparts. The dataset included two predefined image categories: western blot images (27 authentic and 27 AI-manipulated) and subcutaneous xenograft tumor images (25 authentic and 25 AI-manipulated). Each AI-generated image was designed to match a corresponding authentic image in a 1:1 ratio.

## Image collection and AI-manipulated image preparation

Authentic images were collected from the original study materials and used as the reference set. For each authentic image, a matched AI-manipulated counterpart was produced using GPT Image 2. Prompts instructed the model to generate an image with a result trend opposite to that of the original image. This procedure was applied separately to western blot and tumor images, yielding a balanced set of authentic and AI-manipulated images within each image category. The generation workflow was intentionally adversarial and proof-of-concept in nature. For each authentic source image, multiple attempts could be made until a visually plausible manipulated counterpart with an opposite apparent result trend was obtained. Outputs judged to be clear failures (eg, inability to preserve the image modality, inability to reverse the apparent trend, or obvious generation defects) were not retained. Because this workflow was designed to assemble a benchmark of successful manipulations, the final dataset should be understood as a curated set of successful adversarial examples rather than a representative sample of arbitrary model outputs.

## AI-detector evaluation

Three publicly available AI-image detection tools were evaluated independently: Illuminarty, Is It AI, and AI or Not. Each image was submitted to all three detectors, and detector outputs were recorded as continuous scores representing the probability or likelihood that the image was AI-generated. Because score calibration varied across tools, detector performance was evaluated primarily using receiver operating characteristic (ROC) analyses rather than a single fixed threshold.

Performance was assessed in the full dataset and separately in the western blot and tumor subsets. ROC curves were generated using ground-truth image status as the binary outcome and detector score as the predictor. Discriminative performance was summarized by the area under the ROC curve (AUC) with 95% confidence intervals. Pairwise differences in AUC between detectors were assessed using DeLong’s test. For each detector and analysis set, the optimal classification threshold was determined using the Youden index. Detector scores were then converted to binary predictions using the ROC-derived threshold and score direction, and confusion matrices were constructed to obtain true-positive, false-positive, true-negative, and false-negative counts. Sensitivity, specificity, positive predictive value, negative predictive value, accuracy, F1 score, Youden index, and positive and negative likelihood ratios were calculated from these counts.

## Human-reader evaluation

Twenty-four human readers from Peking Union Medical College and Sun Yat-sen University School of Medicine participated in the reader study. All readers held PhD degrees and had more than 5 years of experience in basic biomedical research. Reader assessment was conducted using a questionnaire-based design. For each reader, 50 images were randomly sampled from the full 104-image dataset, including 25 western blot images and 25 subcutaneous xenograft tumor images. Within each image type, authentic and AI-manipulated images were sampled at an approximately 1:1 ratio. Questionnaire generation and image order were randomized for each participant. The study was conducted in a single-blind manner: readers were not informed of the number or proportion of authentic and AI-manipulated images presented. Responses were mandatory for all items. Readers were asked to classify each image as authentic or AI-manipulated.

Reader performance was assessed by comparing each reader’s classification with the reference labels. For each participant, sensitivity, specificity, positive predictive value, negative predictive value, accuracy, F1 score, Youden index, and positive and negative likelihood ratios were calculated. Participant-level performance was summarized as mean ± SD across readers. In addition, pooled reader responses across all reading events were reported as a descriptive summary of overall human performance. Because each participant evaluated multiple images and each image was assessed by multiple participants, these pooled responses were not statistically independent and were not treated as a basis for formal inference. A majority-vote analysis was also performed as an image-level aggregate using all available reader responses for each image, with tied votes treated as missing.

## Statistical analysis

All analyses were performed in R (version 4.3.0) using the pROC, ggplot2, and dplyr packages. Detector score distributions for authentic and AI-manipulated images were compared using two-sided Wilcoxon rank-sum tests across the full dataset and within each image type. ROC analyses were performed separately for the full dataset and for each predefined subgroup. All analyses used complete-case data for the relevant outcome–predictor pair. A two-sided P value < 0.05 was considered statistically significant, and 95% confidence intervals were reported where appropriate.

In addition to the Youden-optimized threshold, we also evaluated detector performance at two other prespecified operating points: the detector’s default threshold of 0.5 and a threshold selected to achieve specificity of approximately 95%. This was done to examine how diagnostic performance changed under different operating conditions and to provide threshold-dependent measures relevant to potential screening applications.

For each threshold, we calculated sensitivity, specificity, positive predictive value (PPV), negative predictive value (NPV), and accuracy. We additionally calculated the F1 score, Youden index, positive likelihood ratio (LR+), and negative likelihood ratio (LR-). False-positive rate and false-negative rate were defined as 1-specificity and 1-sensitivity, respectively.

PPV and NPV for detector analyses were calculated based on the observed study prevalence of 50%, reflecting the balanced design of the image dataset.

## Human-reader evaluation

Twenty-four human readers from Peking Union Medical College and Sun Yat-sen University School of Medicine participated in the reader study. All readers held PhD degrees and had more than 5 years of experience in basic biomedical research. Reader assessment was conducted using a questionnaire-based design. For each reader, 50 images were randomly sampled from the full 104-image dataset, including 25 western blot images and 25 subcutaneous xenograft tumor images. Within each image type, authentic and AI-manipulated images were sampled at an approximately 1:1 ratio. Questionnaire generation and image order were randomized for each participant. The study was conducted in a single-blind manner: readers were not informed of the number or proportion of authentic and AI-manipulated images presented. Responses were mandatory for all items. Readers were asked to classify each image as authentic or AI-manipulated.

Reader performance was assessed by comparing the reader's output with the reference labels. For each participant, sensitivity, specificity, positive predictive value, negative predictive value, accuracy, F1 score, Youden index, and positive and negative likelihood ratios were calculated. Participant-level performance was summarized as mean ± SD across readers. In addition, all reader responses were pooled to estimate overall human performance. A majority-vote analysis was also performed at the image level using all available reader responses for each image, with tied votes treated as missing.

## Statistical analysis

All analyses were performed in R (version 4.3.0) using the pROC, ggplot2, and dplyr packages. Detector score distributions for authentic and AI-manipulated images were compared using two-sided Wilcoxon rank-sum tests across the full dataset and within each image type. ROC analyses were performed separately for the full dataset and for each predefined subgroup. All analyses used complete-case data for the relevant outcome–predictor pair. A two-sided P value < 0.05 was considered statistically significant, and 95% confidence intervals were reported where appropriate.

All analyses were performed in R (version 4.3.0) using the pROC, ggplot2, and dplyr packages. Because each manipulated image was derived from a matched authentic image, both unpaired and paired analytical approaches were considered. ROC analyses were performed separately for the full dataset and for each predefined subgroup, using ground-truth image status as the binary outcome and detector score as the predictor. Discriminative performance was summarized by the area under the ROC curve (AUC) with 95% confidence intervals. To assess robustness, bootstrap mean AUC values and bootstrap 95% confidence intervals were additionally calculated. Pairwise differences in AUC between detectors were assessed using DeLong’s test for correlated ROC curves.

To explicitly account for the paired structure of the dataset, detector scores assigned to AI-manipulated images were compared with those assigned to their matched authentic counterparts using paired Wilcoxon signed-rank tests. These analyses were conducted overall and separately within the western blot and tumor subsets. For each paired comparison, the V statistic, two-sided P value, and median paired difference were recorded.

Threshold-dependent diagnostic performance was evaluated at three operating points for each detector: the default threshold (0.5), the Youden-optimized threshold, and a threshold chosen to achieve specificity of approximately 95%. For each operating point, confusion-matrix-based metrics were calculated, including sensitivity, specificity, PPV, NPV, accuracy, F1 score, Youden index, LR+, and LR−.

Detector score distributions between authentic and AI-manipulated images were also compared using two-sided Wilcoxon rank-sum tests across the full dataset and within each image type. All analyses used complete-case data for the relevant outcome-predictor pair. A two-sided P-value < 0.05 was considered statistically significant, and 95% confidence intervals were reported where appropriate.

**Exact prompt templates fed into GPT Image 2**

Real western-blot result fed into the ChatGPT Image 2.0

Generate a modified version of the reference image with these rules:

**1. Trend:**

For example: gradually increasing from left to right / small in the first row and large in the third row.

**2. Morphology:**

For example: thick at both ends and thin in the middle / with a defect in the middle / one side thick and the other side thin

**3. Background & style:**

For example: background depth, noise, lighting and shadows, clarity, water stains/rust spots/bloodstains

**4. Constraints:**

For example: All labels, arrows, and layout must be IDENTICAL to the reference image/ No new text, no layout changes/ Must look realistic, allow unevenness, inhomogeneity, and minor artifacts.

5. **Generation parameters:**

For example: Mode: image-to-image / inpainting (as needed); Strength: 0.65-0.85 (adjust based on change magnitude); Guidance scale: 7.5-8.5; Seed: random

Paired ChatGPT Image 2.0 Manipulation Image
